# Supplementary material for: Association between oral microbiome and five types of respiratory infections: a two-sample Mendelian randomization study in east Asian population
Source: Front Microbiol. 2024 Apr 10;15:1392473. doi: 10.3389/fmicb.2024.1392473 (PMC11039966; doi:10.3389/fmicb.2024.1392473)
Supplement: Supplementary file 1 [file Data_Sheet_1.zip › Supplementary Materials/MR plots of saliva/Tonsillitis/s__Streptococcus_sp000235485_mgs_302/forest.pdf]

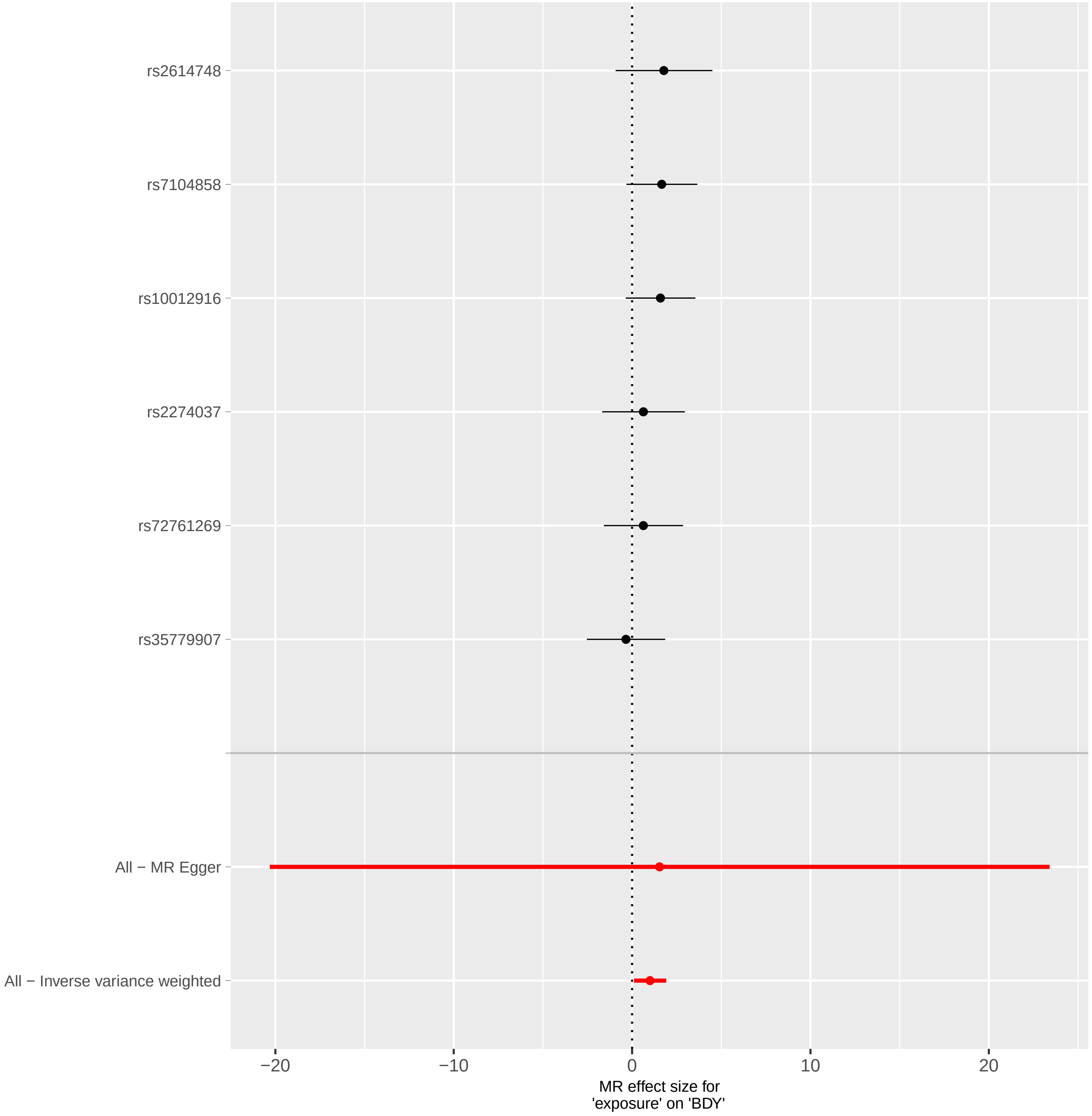
rs2614748
rs7104858
rs10012916
rs2274037
rs72761269
rs35779907
All − MR Egger
All − Inverse variance weighted
−20
−10
0
10
20
MR effect size for
'exposure' on 'BDY'
